# Trends in vitamin D supplement use in a general female and breast cancer population in Ireland: A repeated cross-sectional study

**DOI:** 10.1371/journal.pone.0209033

**Published:** 2018-12-13

**Authors:** J. M. Madden, M. J. Duffy, L. Zgaga, K. Bennett

**Affiliations:** 1 Population Health Sciences Division, Royal College of Surgeons in Ireland, Dublin, Ireland; 2 UCD School of Medicine, Conway Institute of Biomolecular and Biomedical Research, University College Dublin, Dublin, Ireland; 3 Department of Public Health and Primary Care, Trinity College Dublin, Dublin, Ireland; Turun Yliopisto, FINLAND

## Abstract

**Background:**

Vitamin D has been linked with improved survival after breast cancer diagnosis but little is known about prescribing rates. This study investigates trends in vitamin D supplement use in both a general female and breast cancer population.

**Methods:**

Women with a breast cancer diagnosis were identified from the National Cancer Registry of Ireland (n = 19870). Women who had any vitamin D claim between 2005 and 2011 were identified from pharmacy claims data (n = 8556). Prevalence rates were calculated as a proportion of all eligible women and by age (< 55 years, ≥ 55 years). Poisson regression was used to compare rates of vitamin D prescribing across years (risk ratio (RR), 95% CI).

**Results:**

There was a statistically significant increase in women with a claim for vitamin D between 2005–2011, with the largest increase among breast cancer patients aged ≥ 55 years (RR = 2.26; 95% CI, 2.11–2.42).

**Conclusion:**

This may have significant public health implications if associations between vitamin D and improved breast cancer survival prove to be causal.

## Introduction

Several studies examining serum 25-hydroxyvitamin D [25(OH)D] levels have shown significant protective effects of high levels on breast cancer survival [1–4]. For example, a recent meta-analysis of 64 prospective studies found that, among breast cancer patients (8 studies), higher circulating 25(OH)D levels measured at time of diagnosis were associated with a significant 25% reduction in breast cancer mortality [3]. Compounded by the high rates of vitamin D deficiency among the general population [5] and particularly among cancer patients [6], vitamin D supplementations could have a major impact on breast cancer outcomes.

Although conclusive evidence is still largely lacking, dramatic increases in requests for 25(OH)D tests reflects the recent reignited interest in vitamin D which seems to be driven by both health professionals and the general public [5, 7, 8]. However, it is not known whether the use of vitamin D supplements has also increased, particularly among breast cancer patients. For example, vitamin D is often now prescribed with aromatase inhibitors (AI) as a potential treatment for AI-associated bone loss [9]. Thus, the aim of this study was to evaluate trends in vitamin D pharmacy claims in women over time, in both a breast cancer and general population cohort, and to determine if the initial prescribed dose among new vitamin D users increased over the same period. Additionally, we investigated trends in AI pharmacy claims and examined if vitamin D claims among AI users increased over time.

## Methods

The study utilised data from Ireland’s Health Service Executive (HSE) Primary Care Reimbursement Services (PCRS) pharmacy claims database. The PCRS is responsible for reimbursement of claims made under the General Medical Services (GMS) scheme. The scheme covers approximately one third (1.4 million) of the Irish population with enrolment to the scheme based on means test with upper thresholds for eligibility based on weekly income and age. As a result, those on the scheme tend to be older and have a lower socioeconomic [10]. It provides universal healthcare, including free medications, to those on the scheme. Claims are usually made on a monthly basis where we assume a month has 28 days. The data has been linked to the National Cancer Registry of Ireland (NCRI) [10] which gathers data on diagnosed cancer cases.

All women (aged ≥ 16 years) with and without a diagnosis of breast cancer who had a vitamin D claim (WHO Anatomical Therapeutic Chemical (ATC) Classification System: A11CC (vitamin D) and relevant codes A12AX (calcium and vitamin D combination)) dispensed between 2005–2011 were extracted. This enabled us to separately explore those with a vitamin D claim within (i) total GMS-eligible population and (ii) GMS-eligible breast cancer patients. Data were additionally stratified by age (< 55 years and ≥ 55 years). These cut-points were chosen to identify timing of the menopause as many women are concerned about potentially deteriorating bone health post-menopause and may start using calcium and vitamin D supplements. For the GMS-eligible breast cancer cohort, we also extracted relevant AI (ATC: L02BG) and tamoxifen (ATC: L02BA01) claims. This enabled us to calculate the percentage of breast cancer cases with any AI claim also receiving any vitamin D claim in the same year. Prevalence’s of vitamin D supplementation per 1000 GMS female population and breast cancer population were calculated as a proportion of all eligible women, based on annual HSE-PCRS reports [11] and the NCRI database. Poisson regression was used to compare rates of prescribing across years.

A new user was defined as not having received any claim for vitamin D in the previous year. The initial vitamin D claim (and dose) was identified from the new users in the GMS-eligible female population. For the breast cancer cohort, the first vitamin D claim after their diagnosis was considered their initial claim for the purpose of the study. To determine if women changed the strength of vitamin D over time, the dose intake was categorised into four groups (1–399, 400–799, 800–1199, 1200+ IU/day). The percentage of women switching between dose categories was calculated for the general and breast cancer cohorts. All analysis was conducted using R statistical software package.

The use for research of anonymised data held by the NCRI and PCRS is covered by the Health (Provision of Information) Act 1997 and does not require patient consent.

## Results

**Fig 1:**
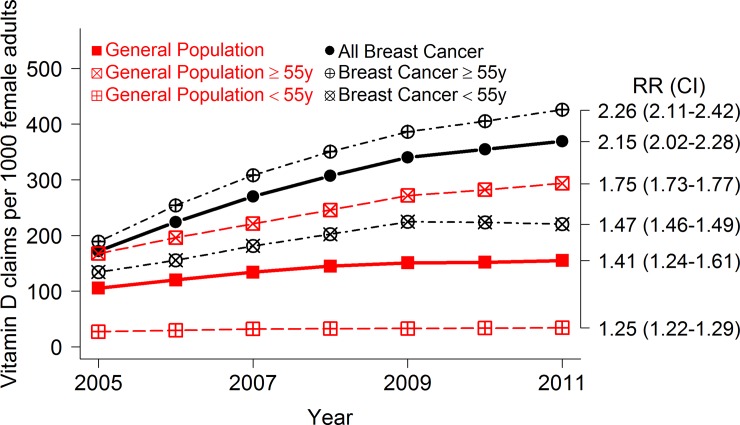
Overall trends in rate of women in receipt of any vitamin D per 1000 women for (i) GMS eligible national population and (ii) GMS breast cancer patients for years 2005–2011 with stratification by age group. Relative risks (RR) along with confidence intervals (CI) are also presented comparing 2011 to 2005 where the referent group for each RR is the group specific level in 2005.

During 2005, vitamin D supplements were dispensed to 55,250 women in the GMS female population, representing a prevalence rate of 105.4/1000 (95% CI 104.5–106.3) per eligible population, and to 1,285 breast cancer patients, representing 172.1/1000 (95% CI 162.7–181.5) breast cancer eligible population. By 2011, these figures had risen to 155.1/1000 (95% CI 154.1–156.0) and 369.3/1000 (95% CI 359.7–378.8) respectively. Fig 1 provides overall trends in rate of women in receipt of any vitamin D (per 1000 women for general and breast cancer cohorts) and risk ratios (RR) comparing 2011 to 2005 and by age group. Across all age and cohorts there was a statistically significant increase in rates for vitamin D over time. The rate of vitamin D doubled for the breast cancer patients between 2005 and 2011, and increased by 50% for the GMS general female population. Stratification by age (< 55 years and ≥ 55 years) highlighted that for both cohorts, women aged ≥ 55 years had much higher prescribing rates of vitamin D compared to those aged < 55 years (Fig 1). The largest increase was among breast cancer patients aged ≥ 55 years with a statistically significant 2.3- fold increase in women with a claim (RR = 2.26; 95% CI, 2.11–2.42, Fig 1). The smallest increase was among the general female population aged < 55 years (RR = 1.25; 95% CI, 1.22–1.29, Fig 1). The vast majority (98%) of all vitamin D claims for both the general female population and breast cancer cohort were for a vitamin D and calcium combination product (ATC: A12AX). The median initial starting dose for new vitamin D users over the seven years for all groups remained constant at 857IU/day equivalent to a monthly claim of 60 tablets at strength 400IU. The percentage of women whose claims remain in the same IU/day dose category was 30% and the percentage of women who had claims from any two categories was 54% with the remainder having a claim from any three or all of the categories. We also determined persistence rates, 75% of those with a claim for vitamin D, had an additional claim between 6–12 months after their first vitamin D claim.

The number of breast cancer patients by vitamin D/tamoxifen/AI use and percentage of patients with any AI claim who also had any vitamin D claim in the same year is provided in Table 1. Overall, there was no increase in claims for AIs from 2005–2011 but the percentage of patients with an AI claim who also had any vitamin D claim in the same year increased from 36% to 68% (RR = 1.87; 95% CI, 1.74–2.29). Claims of tamoxifen decreased from 31% to 18% over time (RR = 0.59; 95% CI, 0.56–0.62) while the number the percentage of patients with a tamoxifen claim who also had any vitamin D claim in the same year increased from 19% to 34% (RR = 1.87; 95% CI, 1.67–2.10). Among patients who were not on endocrine therapy, vitamin D use also increased over the time period (results not shown, RR = 1.77; 95% CI, 1.53–2.05).

**Table 1:** NCRI-PCRS prescribing data: Number of female breast cancer patients with breakdown by vitamin D/tamoxifen/aromatase inhibitors (AI) users and the percentage of AI users who had a vitamin D claim.

Year	Vitamin D	Tamoxifen	Aromatase Inhibitors (AI)	n (%) of AI users with a vitamin D claim	n (%) of Tamoxifen users with a vitamin D claim
**2005 (n = 7465)**	1285 (17%)	2351 (31%)	2358 (32%)	852 (36%)	434 (19%)
**2006 (n = 8752)**	1962 (22%)	2302 (26%)	3151 (36%)	1373 (44%)	498 (16%)
**2007 (n = 10174)**	2749 (27%)	2216 (21%)	3848 (38%)	1940 (50%)	562 (15%)
**2008 (n = 11678)**	3588 (31%)	2244 (19%)	4369 (37%)	2494 (57%)	615 (25%)
**2009 (n = 13060)**	4443 (34%)	2396 (18%)	4811 (37%)	2958 (61%)	733 (31%)
**2010 (n = 14381)**	5100 (35%)	2634 (18%)	4985 (35%)	3192 (64%)	854 (32%)
**2011 (n = 15601)**	5761 (37%)	2899 (18%)	5100 (33%)	3450 (68%)	999 (34%)

n (%), ATC codes: Vitamin D = A11CC/A12AX; Aromatase inhibitors (AI) = L02BG, Tamoxifen = L02BA01

## Discussion

This is the first study to report on trends in vitamin D pharmacy claims in a breast cancer cohort. Our main finding was that there was 2.3 fold increase in vitamin D claims for women aged ≥ 55 years with a diagnosis of breast cancer over the seven year period. We did not have information on the indication for vitamin D, but as the majority of the claims were for vitamin D and calcium combination product (rather than vitamin D only agent), one possibility is that it was prescribed for the prevention of possible bone loss. This is supported by our findings which show a significant increase in vitamin D use among those with any AI claim. Women taking AIs are at increased risk of bone fracture and as a result more likely to be prescribed calcium and vitamin D based agents [9]. We suggest that the most likely reason that prescribers are prescribing vitamin D is to promote calcium absorption which has in turn been linked with improved bone health, irrespective of the evidence for or against bone health [12]. Another possible indication could be for vitamin D deficiency with recent increases in vitamin D testing [13].

The large increase in rates of vitamin D use, as illustrated in this study, is likely to have significant implications on our health systems and clinical practice, and potentially cancer outcomes if associations between vitamin D and breast cancer prognosis prove to be causal [14]. As we have highlighted, there is strong epidemiological and lab based evidence to suggest that serum vitamin D levels (not supplements per se) are associated with improved breast cancer survival [3, 15, 16]. Regarding supplements, we have recently found that de novo vitamin D supplement use post-diagnosis was associated with a statistically significant 20% reduction in breast cancer mortality compared to non-users [17]. In the meantime however, as many clinicians are now increasingly including blood tests to measure vitamin D concentrations as part of routine laboratory work and, then subsequently prescribing vitamin D supplements, there is excess burden across the health system in terms of time, labour and most significantly, the financial ramifications from testing that requires further justification [7, 13, 18].

Unfortunately, until findings from ongoing randomised controlled trials (RCT) are available, we will not definitively know whether vitamin D supplementation is of benefit to patients with chronic diseases, including breast cancer [7]. Moreover, there have also been suggestions that extremely high doses can pose potential risks such as increased risk of fractures and falls but the evidence is still not clear [19].

Although the majority of the claims were for vitamin D and calcium combination product, both vitamin D alone and vitamin D/calcium combination are both covered by the GMS scheme. Our focus here is on vitamin D and breast cancer patients and we do not suggest that calcium prevents breast cancer progression. There has been recent evidence to suggest the limited use of calcium for bone growth [12]. Interestingly, evidence from the Women’s Health Initiative clinical trial of over 36,000 postmenopausal women which examined the effects of calcium and vitamin D supplementation versus placebo on risk fractures found no significant difference in GI side-effects including constipation and bloating between calcium and vitamin D users compared to the placebo group [20]. However, we agree that the use of calcium is not without adverse effects and adherence has been shown to be an issue.

The major strength of the study is the access to the large, linked high quality, reliable and complete national cancer and national pharmacy claims data. This is the first study at a national level that we are aware of, to report on overall trends of vitamin D supplementation use among breast cancer patients. However, as access to the GMS scheme is means tested, the GMS population used in our study tends to be older and more socioeconomically deprived, yet, it is still representative of a comprehensive nationwide population that covers a long time frame. Unfortunately however, we only have linked cancer registry and pharmacy claims data linked up to 2011. We had no information on the indication of vitamin D use. We also do not know about vitamin D supplements purchased over-the-counter, although it is less likely to have occurred in those eligible for the GMS scheme as all medicines would have been mostly free under the scheme.
